# Perioperative glucocorticoid administration for prevention of systemic organ failure in patients undergoing esophageal resection for esophageal carcinoma

**DOI:** 10.1590/S1516-31802006000200013

**Published:** 2006-03-02

**Authors:** Antônio Marcos Raimondi, Hélio Penna Guimarães, José Luiz Gomes do Amaral, Patrícia Helena Rocha Leal

**Keywords:** Esophagectomy, Methylprednisolone, Multiple organ failure, Review literature, Meta-analysis, Esofagectomia, Metilprednisolona, Falência de múltiplos órgãos, Literatura de revisão, Metanálise

## Abstract

**CONTEXT AND OBJECTIVE:**

Preoperative glucocorticoid administration has been proposed for reducing postoperative morbidity. This is not widely used before esophageal resection because of incomplete knowledge regarding its effectiveness. The aim here was to assess the effects of preoperative glucocorticoid administration in adults undergoing esophageal resection for esophageal carcinoma.

**SEARCH STRATEGY:**

Studies were identified by searching the Cochrane Controlled Trials Register, MEDLINE, EMBASE, Cancer Lit, SCIELO and Cochrane Library, and by manual searching from relevant articles. The last search for clinical trials for this systematic review was performed in December 2004.

**SELECTION CRITERIA:**

This review included randomized studies of patients with potentially resectable carcinomas of the esophagus that compared preoperative glucocorticoid administration with placebo.

**DATA COLLECTION AND ANALYSIS:**

Data were extracted by the same reviewers, and the trial quality was assessed using Jadad scoring. Relative risk and weighted mean difference with 95% confidence limits were used to assess the significance of the difference between the treatment arms.

**RESULTS:**

Four randomized trials involving 146 patients were found. There were no differences in postoperative mortality, sepsis, anastomotic leakage, hepatic and renal failure between the glucocorticoid and placebo groups. There were fewer postoperative respiratory complications (p = 0.005) and multiple postoperative complications (p = 0.004) and lower postoperative plasma interleukin-6 levels (p = 0.00001) with preoperative glucocorticoid administration. There was a higher postoperative PaO_2_/FiO_2_ ratio (p = 0.0001) with preoperative glucocorticoid administration.

**CONCLUSION:**

Prophylactic administration of glucocorticoids is associated with decreased postoperative complications.

## RESUMO

**CONTEXTO E OBJETIVO:**

A administração de glicocorticóides tem sido proposta para reduzir a morbidade após operações, porém, não é largamente usada antes da ressecção do esôfago devido ao fato de não haver ainda conhecimentos suficientes sobre sua eficácia. O objetivo do trabalho é verificar os efeitos da administração pré-operatória de glicocorticóides em pacientes submetidos a esofagectomia por carcinoma de esôfago.

**ESTRATÉGIA DE PESQUISA:**

As fontes utilizadas foram: Medline, Embase, Cancerlit, SciELO, Base de Dados de Ensaios Clínicos Controlados da Colaboração Cochrane e busca manual de referências. O término da pesquisa ocorreu em dezembro de 2004.

**CRITÉRIOS DE SELEÇÃO:**

Estudos randomizados de pacientes com carcinoma esofágico que compararam glicocorticóide com placebo administrados antes das esofagectomias.

**ANÁLISE E COLETA DE DADOS:**

Os dados foram coletados pelos mesmos revisores e a qualidade dos estudos foi avaliada usando-se o escore de Jadad. A metanálise foi realizada utilizando-se o risco relativo e diferença de média ponderada entre tratamento e placebo (intervalos de confiança de 95%).

**RESULTADOS:**

Quatroensaios envolvendo 146 pacientes foram localizados. Não ocorreram diferenças na mortalidade pós-operatória e nas incidências de sepse, deiscência de anastomose e insuficiências renal e hepática entre glicocorticóide e placebo. Houve decréscimo na incidência de complicações respiratórias pós-operatórias (p = 0,005), múltiplas complicações (p = 0,004) e níveis plasmáticos de interleucina-6 (p = 0,00001) nos pacientes que receberam glicocorticóide pré-operatório. Houve incremento na relação PaO_2_/FiO_2_ pós-operatória (p = 0,0001) no grupo que recebeu glicocorticóide pré-operatório.

**CONCLUSÃO:**

A administração pré-operatória de glicocorticóide foi associada com um decréscimo nas complicações pós-operatórias.

## INTRODUCTION

Major surgery induces a severe inflammatory response, such as raised levels of adrenocorticotropic hormones and acute-phase reac- tants.^1-3^ Other researchers have reported that a severe inflammatory response may be related to the development of severe postoperative complications.^2-3^ Esophagectomy for esophageal carcinoma is one of the most invasive procedures among gastrointestinal operations and is still associated with high morbidity due to postoperative complications.^4-7^ Pulmonary complications in particular can be fatal, and postoperative hypoxemia is a major cause of anastomotic leakage.^2,3^

Increasing attention has been given towards modulating these postoperative deleterious responses. It has been reported that glucocorticoids have been effective in suppressing the inflammatory response secondary to sepsis and other stress-related disease states.^1,8,9^ A few studies have reported that preoperative methylprednisolone administration attenuates the metabolic response, protects against elevation of proinflammatory cytokine levels and enables adequate postoperative oxygenation following esophagectomy.^2,3,10,11^ Glucocorticoids are not widely used before esophageal resection because of incomplete knowledge regarding their effectiveness and possible adverse effects.

On this basis, the aim of the present systematic review was to investigate the prophylactic effect of perioperative administration of glucocorticoids on postoperative organ dysfunction and morbidity, in patients undergoing esophageal resection due to carcinoma.

## METHODS

This systematic review included randomized or *quasi*-randomized studies on patients with potentially resectable carcinomas of the esophagus (of any histological type) that compared preoperative glucocorticoid administration with placebo. Studies were identified by searching the Cochrane Controlled Trials Register, Medline (1966 - 2004), Embase (1988 - 2004), CancerLit (1993 - 2004), SciELO (1993 - 2004) and Cochrane Library. In addition, manual searching from relevant articles and personal files was included. We also contacted manufacturers and researchers. There were no language restrictions.

Data were extracted by the same reviewers, and the trial quality was assessed using Jadad scoring.^12^ The inclusion of a study depended on the evaluation of the randomization. The most important criterion for the classification was the allocation concealment, which needed to have been maintained until the time of the intervention.^13^ The data were collected using software from the Cochrane Collaboration: Review Manager, Version 4.2.3 for Windows, Oxford (UK).

The primary outcome of interest was overall mortality. Other variables examined were: anastomotic leakage, postoperative respiratory complications, renal failure, hepatic failure, multiple postoperative complications (two or more complications per patient), postoperative plasma levels of interleukin-6, postoperative PaO_2_/FiO_2_ ratio and postoperative hospital stay.

Sensitivity analysis was planned in order to explore sources of heterogeneity, where heterogeneity existed. The factors hypothesized *a priori* included quality of study and biologically effective corticosteroid dose. Relative risk (RR) and weighted mean difference (WMD) with 95% confidence limits were used to assess the significance of the difference between the treatment arms.^13,14^

## RESULTS

Four randomized controlled clinical trials comparing glucocorticoid (methylprednisolone) with placebo were found in the literature search. Thus, 146 patients were divided into two groups: the placebo group consisted of 77 patients who received injections of saline solution, and the intervention group consisted of 69 patients to whom methylprednisolone was administered intravenously, before the induction of anesthesia. The characteristics of the clinical trials included in the systematic review are shown in Table 1.

**Table 1:** Clinical trials included in this systematic review and meta-analysis

Authors	sample	intervention	placebo	Jadad scores
Matsutani et al., 1998^10^*	33	14	19	3
Sato et al., 2002^11^*	66	33	33	5
Takeda et al., 1997^2^†	30	15	15	3
Takeda et al., 2003^3^*	17	7	10	3

* *Methylprednisolone: 10 mg/kg before induction of anesthesia*;

† *Methylprednisolone: 30 mg/kg before induction of anesthesia.*

There were no differences in postoperative mortality, anastomotic leakage and postoperative hospital stay between the glucocorticoid and placebo groups.

There were fewer postoperative respiratory complications (RR = 0.23; 95% confidence interval, CI: 0.08 to 0.65; p = 0.005) and multiple postoperative complications (RR = 0.34; 95% CI: 0.16 to 0.71; p = 0.004), and lower postoperative plasma levels of interleukin-6 (WMD = –374.72; 95% CI: –452.99 to –296.45; p = 0.00001) with preoperative glucocorticoid administration. There was a higher postoperative PaO_2_/FiO_2_ ratio (WMD = 6.43; 95% CI: 3.16 to 9.70; p = 0.0001) with preoperative glucocorticoid administration.

A summary of the meta-analysis results for each variable is presented in Tables 2 and 3, with the numbers of studies included, the numbers of participants, and the results from the heterogeneity and overall effect tests.

**Table 2:** Discrete data: summary of this meta-analysis results

Postoperative outcomes	studies	participants	statistical methods	effect size	test of heterogeneity	test of overall effect
Mortality	4	128	RR (random), 95% CI	0.20 [0.01, 3.85]	Not applicable	Z = 1.07 p = 0.29
Multiple complications	3	129	RR (random), 95% CI	0.34 [0.16, 0.71]	Chi-squared 0.72 df = 2 p = 0.70	Z = 2.85 p = 0.004
Sepsis	3	129	RR (random), 95% CI	0.43 [0.11, 1.64]	Chi-squared 0.62 df = 2 p = 0.73	Z = 1.23 p = 0.22
Respiratory complications	4	146	RR (random), 95% CI	0.23 [0.08, 0.65]	Chi-squared 0.74 df = 2 p = 0.69	Z = 2.78 p = 0.005
Hepatic failure	2	96	RR (random), 95% CI	0.39 [0.10, 1.57]	Chi-squared 0.01 df = 1 p = 0.92	Z = 1.33 p = 0.18
Renal failure	2	96	RR (random), 95% CI	0.81 [0.34, 1.92]	Chi-squared 0.34 df = 1 p = 0.56	Z = 0.47 p = 0.64
Anastomotic leakage	3	113	RR (random), 95% CI	0.35 [0.06, 2.21]	Chi-squared 0.23 df =1 p = 0.63	Z = 1.12 p = 0.26

*RR = relative risk; CI = confidence interval; df = degrees of freedom.*

**Table 3:** Continuous data: summary of the meta-analysis results

Postoperative outcomes	studies	participants	statistical methods	effect size	test of heterogeneity	test of overall effect
PaO_2_/FiO_2_ ratio immediate PO	2	47	WMD (random), 95% CI	3.05 [-6.18, 12.27]	Chi-squared 4.58 df = 1 p = 0.03	Z = 0.65 p = 0.52
PaO_2_/FiO_2_ ratio on POD1	2	47	WMD (random), 95% CI	2.38 [-15.55, 20.31]	Chi-squared 32.01 df = 1 p < 0.00001	Z = 0.26 p = 0.79
PaO_2_/FiO_2_ ratio on POD2	2	47	WMD (random), 95% CI	13.57 [7.40, 19.74]	Chi-squared 3.45 df = 1 p = 0.06	Z = 4.31 p < 0.0001
PaO_2_/FiO_2_ ratio on POD3	2	47	WMD (random), 95% CI	13.62 [9.80, 17.45]	Chi-squared 0.42 df = 1 p = 0.52	Z = 6.98 p < 0.00001
Plasma levels of interleukin-6 on POD1	3	111	WMD (random), 95% CI	-374.72 [-452.99,-296.45]	Chi-squared 8.80 df = 2 p = 0.00001	Z = 9.38 p < 0.00001

*POD = postoperative day; WMD = weighted mean difference; CI = confidence interval; df = degrees of freedom.*

**Figure 1:**
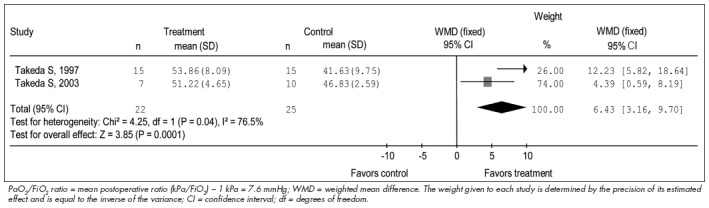
Mean postoperative PaO_2_/FiO_2_ ratio in two studies included in this meta-analysis.

**Figure 2:**
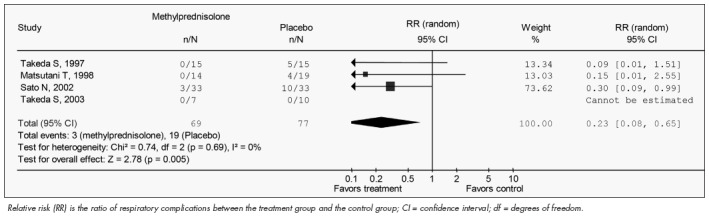
Postoperative respiratory complications in four studies included in this meta-analysis.

The results from the meta-analysis of mean PaO_2_ /FiO_2_ and respiratory complications are shown in Figures 1 and 2, respectively.

## DISCUSSION

There is as yet no agreement on the beneficial effects of corticosteroids in alleviating surgical stress.^1^ This disagreement probably stems from the variability in the drugs used, their dosage and administration schedules, and the nature of the surgical procedures in different studies.

Surgical treatment of thoracic esophageal cancer is one of the most stressful surgical procedures, and the frequency of postoperative organ failure remains very high.^4-7^ Esophageal cancer surgery was therefore selected as one of the most suitable procedures for evaluating the effects of steroids on surgical stress.

Modification of the inflammatory response at an early stage would seem to be very important, because compensatory antiinflammatory responses occur in very quick succession after the inflammatory response. In these studies, therefore, methylprednisolone was administered just before the surgery.

While the anti-inflammatory actions of methylprednisolone are five times as strong as those of cortisol, the actions on electrolyte metabolism are less than half as strong.^11,15,16^ Previous studies on humans and rabbits have demonstrated that methylprednisolone appears in the lung in greater concentrations than prednisolone.^17^ Also, the half-life of methylprednisolone in the blood is 2.8 hours. When methylprednisolone is administered intravenously at a individual dose of 1,000 mg, the maximum blood concentration in healthy adults is about 10 µg/ml, and more than 10 µg/ml.^11^

Postoperative mortality was the same for the methylprednisolone and placebo groups in this meta-analysis. The trials included were relatively small and might not have had sufficient statistical power to detect a clinically significant difference in mortality between the groups.

Administration of methylprednisolone reduced the incidence of multiple and respiratory complications and increased the PaO_2_/FiO_2_ after the first postoperative day. The reduction in multiple complications may have been due in part to a decrease in respiratory complications. Furthermore, the rate of anastomotic leaks was similar in the methylprednisolone and placebo groups. These results suggest that preoperative methylprednisolone administration may be safe for alleviating surgical stress.

The pathophysiology of postoperative organ dysfunction is multifactorial, including additional factors such as hypoxemia, nutrition, pain, type of analgesia, immobilization and surgical expertise.^6,7,18^ The incidence of sepsis and hepatic and renal failures were similar between the methylprednisolone and placebo groups. Nevertheless, multiple postoperative complications were reduced by administering methylprednisolone before surgery. The reason for this apparent discrepancy is the magnification effect that occurs when multiple complications are gathered together.

The present study also showed that preoperative methylprednisolone administration suppressed postoperative increases in the plasma levels of interleukin-6 (IL- 6). IL-6 produced by endothelial cells influences the permeability of cultured endothelial cells to albumin, and anti-IL-6 antibodies can prevent increased endothelial permeability. Studies on heart surgery have suggested that increased levels of proinflammatory cytokines, in particular IL-6, can be correlated with impaired hemodynamics and higher incidence of postoperative complications.^3,8,9^ Therefore, it should be noted that decreased IL-6 levels may be more important for systemic inflammatory responses than for local reactions.

## CONCLUSIONS

The present meta-analysis has shown that the prophylactic administration of methylprednisolone decreased the numbers of postoperative respiratory complications and multiple postoperative complications and the postoperative plasma levels of IL-6. Methylprednisolone pretreatment represents a potentially important biological modifier of perioperative inflammatory responses and organ dysfunction. However, these potential beneficial effects and risks from preoperative methylprednisolone administration should be assessed in large-scale studies.
